# The tRNA-Derived Small RNAs Regulate Gene Expression through Triggering Sequence-Specific Degradation of Target Transcripts in the Oomycete Pathogen *Phytophthora sojae*

**DOI:** 10.3389/fpls.2016.01938

**Published:** 2016-12-22

**Authors:** Qinhu Wang, Tingting Li, Ke Xu, Wei Zhang, Xiaolong Wang, Junli Quan, Weibo Jin, Meixiang Zhang, Guangjin Fan, Ming-Bo Wang, Weixing Shan

**Affiliations:** ^1^State Key Laboratory of Crop Stress Biology for Arid Areas and College of Life Sciences, Northwest A&F UniversityYangling, China; ^2^State Key Laboratory of Crop Stress Biology for Arid Areas and College of Plant Protection, Northwest A&F UniversityYangling, China; ^3^CSIRO AgricultureCanberra, ACT, Australia

**Keywords:** oomycete, *Phytophthora*, tsRNA, RNA silencing, gene regulation

## Abstract

Along with the well-studied microRNA (miRNA) and small interfering RNA (siRNA) is a new class of transfer RNA-derived small RNA (tsRNA), which has recently been detected in multiple organisms and is implicated in gene regulation. However, while miRNAs and siRNAs are known to repress gene expression through sequence-specific RNA cleavage or translational repression, how tsRNAs regulate gene expression remains unclear. Here we report the identification and functional characterization of tsRNAs in the oomycete pathogen *Phytophthora sojae*. We show that multiple tRNAs are processed into abundant tsRNAs, which accumulate in a similar developmental stage-specific manner and are negatively correlated with the expression of predicted target genes. Degradome sequencing and 5′ RLM RACE experiments indicate tsRNAs can trigger degradation of target transcripts. Transient expression assays using GUS sensor constructs confirmed the requirement of sequence complementarity in tsRNA-mediated RNA degradation in *P. sojae*. Our results show that the tsRNA are a class of functional endogenous sRNAs and suggest that tsRNA regulate gene expression through inducing sequence-specific degradation of target RNAs in oomycetes.

## Introduction

Small non-protein-coding RNAs (sRNAs) play important roles in transcriptional and post-transcriptional gene regulation in eukaryotic organisms. Animal and plant sRNAs, particularly microRNA (miRNA) and small interfering RNA (siRNA), have been identified and extensively studied over the last 15 years (Carthew and Sontheimer, 2009). A third class of sRNAs, the Piwi-interacting RNA (piRNA), was identified in animal germlines (Girard et al., 2006; Lau et al., 2006). All these sRNAs employ sequence-specific targeting mechanisms (Carthew and Sontheimer, 2009; Thomson and Lin, 2009).

Recently, a novel class of sRNAs derived from tRNAs, tsRNAs, was discovered independently from *Tetrahymena thermophile* (Pederson, 2010), *Giardia lamblia* (Li et al., 2008), *Saccharomyces cerevisiae* (Thompson et al., 2008), *Arabidopsis* (Hsieh et al., 2009), and HeLa cells (Cole et al., 2009). Further studies on tsRNA accumulation suggested their biological significance. For example, in the filamentous fungus *Magnaporthe oryzae*, tsRNAs were enriched in appressoria in comparison to mycelia (Nunes et al., 2011a). In human cells, the tsRNA tRF-1001 accumulated more abundantly in cancer cell lines than in healthy tissues (Lee Y.S. et al., 2009). In the human parasite euglenoid trypanosomes, *Trypanosoma cruzi*, tsRNAs accumulated specifically in cytoplasmic granules (Garcia-Silva et al., 2010). In human parasitic *G. lamblia*, tsRNA expression is associated with its differentiation (Liao et al., 2014). Much effort also had been directed to understanding the biogenesis of tsRNAs in the past years. A diversity of protein factors have been implicated in tsRNA processing in different organisms, including the RNase T2 family protein Rny1p in yeast (2009), RNase A superfamily protein angiogenin (Fu et al., 2009), RNase III family protein Dicer (Babiarz et al., 2008; Cole et al., 2009), Argonaute (AGO) family proteins (Haussecker et al., 2010), Piwi family proteins Twi12 (Couvillion et al., 2010), and Hiwi2 (Keam et al., 2014), and DNA methyltransferase enzyme Dnmt2 in mammals (Schaefer et al., 2010).

It is documented that tsRNAs were implicated in gene silencing (Haussecker et al., 2010). Unexpectedly, two studies showed that tsRNAs could inhibit protein synthesis without sequence complementarity (Ivanov et al., 2011; Sobala and Hutvagner, 2013). Nonetheless, a 22nt tRNA-derived miRNA has recently been identified which functions like canonical mRNA in human (Maute et al., 2013). More interestingly, a recent study shows that mouse sperm tsRNA could disorder their offspring’s metabolism by targeting promoters of the affected genes (Chen et al., 2016). However, it remains unclear how tsRNAs regulate gene expression and whether they regulate mRNA expression through a sequence-specific mechanism at post-transcriptional level.

Oomycetes, including the Irish potato famine pathogen *Phytophthora infestans*, resemble fungi morphologically but are phylogenetically distant from true fungi (Tyler, 2001). They cause severe damages to crops and natural ecosystems. Virulence variation occurred frequently during the asexual reproduction in the oomycete pathogens *P. sojae* (Rutherford et al., 1985), *P. infestans* (Abu-El Samen et al., 2003), and *Aphanomyces euteiches* (Malvick and Percich, 1998). However, little is known about the underlying molecular basis for such variations. Recent studies show that epigenetic mechanisms, especially the sRNA-directed gene silencing, were implicated in the evolution of virulence in plant pathogens (Kasuga and Gijzen, 2013; Qutob et al., 2013). Intestinally, the origin of gene silencing components of the oomycete pathogens were shown to be evolutionarily unusual (Bollmann et al., 2016), which suggests complexity and roles of sRNAs in these distinctive eukaryotes.

Little is known about the mode of regulation by sRNAs in the oomycete pathogens, although the presence of sRNAs was increasingly evident in these organisms (Vetukuri et al., 2012; Fahlgren et al., 2013; Qutob et al., 2013; Asman et al., 2014). *P. sojae* is a model oomycete species and is a destructive soybean pathogen that causes $1–2 billion losses per year (Tyler, 2007). The genome of this pathogen was sequenced in 2004 and a lot of other genetic resources and tools are available (Tyler et al., 2006; Tyler, 2007; Ye et al., 2011; Fang and Tyler, 2016). Interestingly, some of the *P. sojae* siRNAs were shown to be associated with pathogen virulence (Qutob et al., 2013). We thus used this model species to investigate sRNA-based gene silencing in oomycetes. Using sRNA deep sequencing and RNA degradome analyses, together with the sensor gene expression assays, we identified numerous tsRNAs and generated data supporting a functional role of tsRNAs in gene regulation and developmental control in oomycete pathogens. More significantly, we provided evidence indicating the involvement of a sequence specificity-based RNA degradation in tsRNA-mediated gene regulation.

## Materials and Methods

### *Phytophthora sojae* Strains, Culture, and Soybean Infection Conditions

*Phytophthora sojae* strain P6497 was maintained on 5% CA medium at 25°C in the dark. To prepare *P. sojae*-infected tissues, soybean (*Glycine max* cv Williams) seedlings of 7–10 days were wound-inoculated with *P. sojae* mycelia at hypocotyls as described (Tyler et al., 1995; Shan et al., 2004a). The infected hypocotyls of 48 hpi (hours post inoculation) were collected for expression pattern analysis.

### RNA Isolation

TRIzol (Invitrogen, USA) reagent was used for total RNA isolations following the standard protocol except that RNA precipitation was carried out at –20°C overnight.

### Deep Sequencing of *P. sojae* sRNAs and Degradome Analysis

Equal amounts of RNA from *P. sojae* tissues representing five distinct developmental stages, including oospores, mycelia, sporangia, cysts, and germinated cysts were mixed and sequenced at Beijing Genomics Institute (18–30 nt, accession number: SRR4450568). Sequencing of the 18–50 nt sRNA, the 28–50 nt sRNA, and the degradome of *P. sojae* mycelia were performed at LC Sciences (accession number: SRR4450567, SRR4450570, SRR4450569, respectively).

### Computational and Bioinformatics Analysis

A total of 1,360,211 clean *P. sojae* sRNA reads were obtained by deep sequencing. All reads aligned to the *P. sojae* genome (Tyler et al., 2006) were used for further analysis. Ribosomal RNAs were retrieved from GenBank and gene models generated by the *P. sojae* genome project were also used to filter the short tags. The tsRNAs were identified as shown in Supplemental Figure S1. In brief, eukaryotic tRNA sequences downloaded from Genomic tRNA Database (Chan and Lowe, 2009) were used to extract tRNA-derived sRNA reads. The 75 nt upstream and downstream flanking genomic sequences were extracted for clover-leaf structure screening by RNAshapes (Steffen et al., 2006). We chose the highest MFE (minimum free energy)/length ratio fragment of the corresponding genomic sequences and assembled them to unique sequences by CAP3 (Huang and Madan, 1999) to generate a reference sequences set of tRNA-derived sRNA tags, and the precise tRNA coding regions were defined by feeding the non-redundant reference set to tRNAscan-SE (Lowe and Eddy, 1997). The sRNA sequences were mapped to their references using Bowtie (Langmead et al., 2009) with no mismatch allowed. The generated landscapes were used to determine the sRNA candidates by identification of the peaks in the mapping landscapes via the Base Deep Index (*BDI = floor*[*9×*(*x-x_min_*)/(*x_max_–x_min_*)]) we defined, which reflects the relative coverage of each base along the tRNA sequence. All tsRNA core sequences identified were listed in Supplemental Table S1.

To search for potential target genes of tsRNAs in *P. sojae*, the entire gene models with their 200 bp flanking sequences were searched against tsRNA candidates using NCBI BLAST 2.2.18 (Altschul et al., 1997). Since no information was available on the presence of sequence-specific targeting by tsRNAs in *Phytophthora*, we used relatively less stringent criteria and searched potential target transcripts that have complementary match in local perfect manner with tsRNAs. Only antisense hits (E-value = 10, leading at least 12 bp) were considered as candidate targets. The details of tsRNA-target pairs and the potential function of target genes is shown in Supplemental Tables S2 and S3, respectively. The relationship between tsRNAs and their targets were visualized by Circos (Krzywinski et al., 2009). Digital transcriptome data were retrieved from the *Phytophthora* Transcriptome Database (Ye et al., 2011) and were used to evaluate the expression patterns of *P. sojae* tsRNA targets. Target genes without expression data in the five developmental stages were excluded from the analysis, and the resulting matrix was normalized by both row and column to generate the heatmap using Pearson’s correlation coefficient for pairwise distances. The selected 10 targets for qPCR verifications are listed in Supplemental Table S4. The degradome sequencing reads were mapped to the reference sequence via Bowtie (Langmead et al., 2009).

The re-sequenced *P. sojae* tsRNAs were analyzed using in-house tsRNA annotation pipeline tsRFinder^1^ with its default parameters. All the reference tsRNA sequences were list in Supplemental Table S5.

### Manual Cloning of tsRNAs

For manual cloning of tsRNAs, sRNAs ranging from 15 to 50 nt in size were isolated from the total RNA extracted from mixed *P. sojae* oospores, mycelia, sporangia, cysts, and germinated cysts. After dephosphorylation with Antarctic Phosphatase (NEB, USA), the sRNAs were ligated with 3′ AppDNA adaptor A1 by T4 RNA ligase (NEB, USA) at 14°C overnight, and separated by 15% Urea-PAGE. T4 Polynucleotide Kinase (NEB, USA) was used to add additional 5′-phosphates to the recycled products before subsequent ligation with 5′ RNA adaptor A2 by T4 RNA ligase (NEB, USA). The ligated sRNAs were reverse transcribed with M-MLV (Promega, USA) using 3′ PCR primer P1. The reversely transcribed sRNAs were amplified with primers P1 and P2 and the products were cloned into pGXT vector (Chen et al., 2009) and sequenced at Beijing Genomics Institute. The sRNAs homologous to the tRNAs were regarded as tsRNAs. The adaptors and primers used for sRNA cloning are listed in Supplemental Table S6.

### Northern Blot Analysis

To perform Northern analysis, 20 μg of total RNAs were denatured at 80°C for 10 min and then subjected to gel separation in a 15% urea-denaturing PAGE. sRNAs were electro-blotted onto Amersham Hybond-N^+^ membrane (GE Healthcare, UK) with a semi-dry transfer cell (BioRad, USA) at 3.3 mA/cm^2^ for 20 min. RNA was fixed by a UV-crosslinker (HL-2000 HybriLinker, UVP) at 1200 × 100 μJ/cm^2^ energy for 2 min, followed by baking at 80°C for 2 h. After pre-hybridization with 10 mL hybridization buffer consisting of 1.5 mg Herring Sperm DNA, 7% SDS and 0.2 M Na_2_HPO_4_ for 8 h, DNA oligonucleotides complementary to the tsRNAs (Supplemental Tables S1 and S5) were labeled with [γ-^32^P]-ATP using T4 Polynucleotide Kinase (TaKaRa, China) and added to the hybridization solution. Hybridization was carried out at 42°C for 8 h in a Hybridizer (HL-2000 HybriLinker, UVP). After discarding the hybridization solution, the filter blots were washed twice, each with 30 mL 2X SSC and 0.5% SDS (preheated at 37°C) for 15 min at 37°C. The filter blots were exposed to phosphoimager screens and the signals were detected by scanning with the FLA-7000 image system (Fujifilm, Japan).

### Quantitative RT-PCR Analysis

For real time RT-PCR, 500 ng of total RNA was treated with DNase I (TaKaRa, China) to remove genomic DNA and the cDNA synthesis was performed using PrimeScript^TM^ RT reagent kit according to the manufacturer’s instructions. PCR reactions were performed using 5 μL of a 1:20 dilution of the first-strand cDNA with SYBR Premix Ex Taq^TM^ II according to the manufacturer’s instructions (TaKaRa, China). Expression levels of the tested genes were quantified using an iQ5 Real-Time Cycler (Bio-Rad, USA). The *P. sojae* gene *PsActA* (gene id 108986) was chosen as a reference gene (Zhao et al., 2011). All samples were standardized to the levels in mycelia and the fold changes of expression were calculated using the 2^ΔΔCt^ method. The data were calculated or analyzed by customized R scripts. All the primers used for quantitative RT-PCR analysis are listed in Supplemental Table S6.

### 5′ RLM-RACE Analysis

To perform 5′ RNA ligase -mediated rapid amplification of cDNA ends (5′ RLM-RACE), the poly (A)^+^ RNA was isolated by using Magnetic mRNA Isolation Kit (NEB, UK) from 200 μg total RNA. The cleaved products are uncapped and carry free phosphate, thus enabling direct ligation with an RNA adaptor RA44 by using T4 RNA Ligase (Ambion, USA). The ligation products were extracted by phenol/chloroform and precipitated with glycogen before synthesis of the first-strand of cDNA by using SuperScript II Reverse Transcriptase (Invitrogen, USA). A nested PCR with ExTaq Hot Start Version (TaKaRa, China) was used to detect the degraded products with primers RA44OP/IP and GSP1/GSP2. The amplicons were further confirmed by sequencing. All adaptors and primers used for 5′ RLM-RACE analysis can be found in Supplemental Table S6.

### GUS Sensor Analysis

For TS4WT, TS4MU, TS7WT, and TS7MU, the *GUS* gene was amplified using primer pairs GUSF/GUSR (carrying restriction enzyme sites and tsRNA-binding sites, see Supplemental Table S6) from pCXGUS-P (Chen et al., 2009), double digested with *Xma*l I and *Nsi* I (Promega, USA), and inserted into plasmid pUbIN. The GUS-BS fragments were then released by *Xma* l and *Nsi* I, blunted with *Pfu* DNA polymerase (TaKaRa, China), and inserted into the *Sma* I site of pHAM34H. For TS7WT2 and TS7MU2, the *GUS* gene was amplified and inserted directly into the *Sma* I site of pHAM34H. These GUS sensor expression constructs were transiently expressed in *P. sojae* protoplasts with polyethylene glycol as described (Dou et al., 2008; Fang and Tyler, 2016). Up to 30 μg test plasmid and 10 μg selection marker plasmid were used. GUS activities were determined at 12 h post incubation using histochemical GUS staining (Dou et al., 2008).

## Results

### tsRNA is an Endogenous sRNA in Oomycete Pathogen *Phytophthora sojae*

**FIGURE 1:**
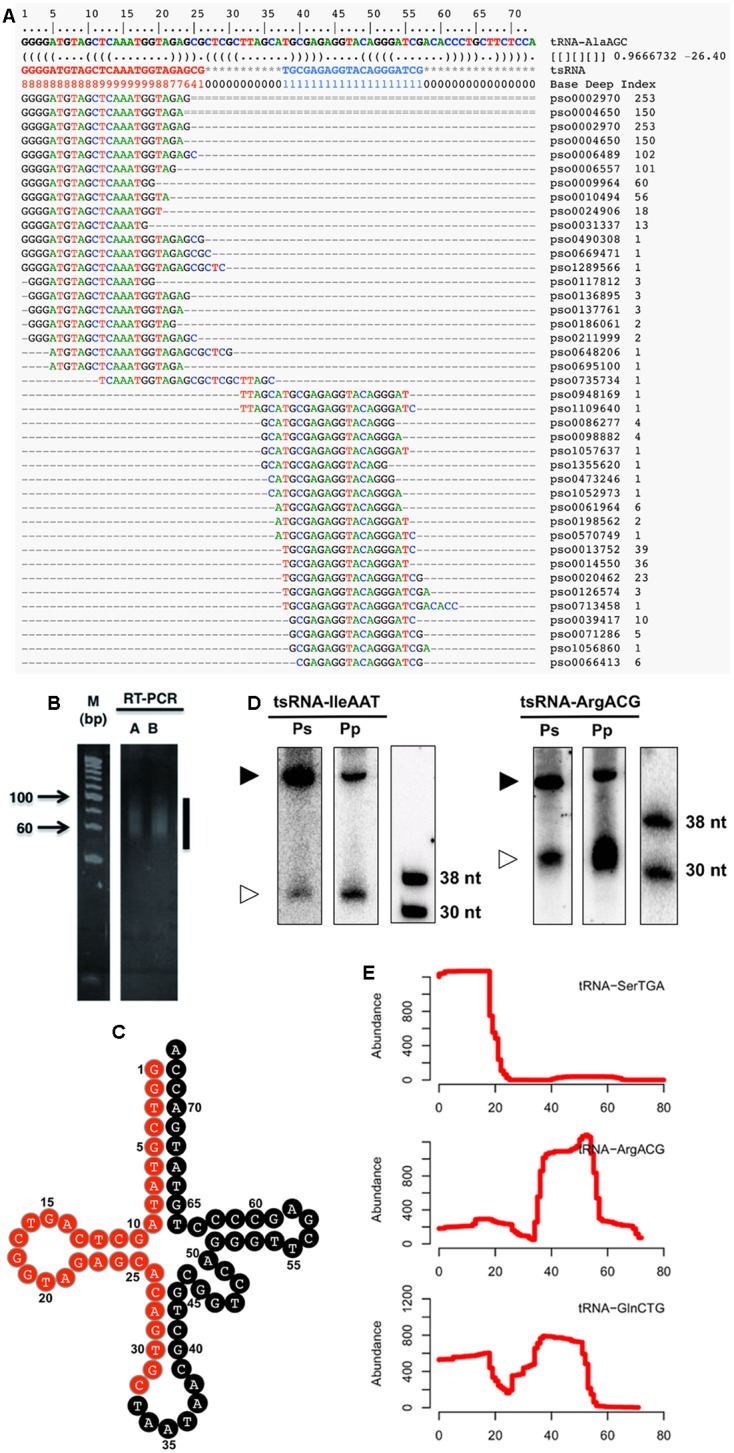
**Identification and verification of *P. sojae* tsRNAs. (A)** Alignment of tsRNA-AlaAGC reads to its cognate tRNA. The first two lines indicate the position of tRNA. The third line is the tRNA sequence. The RNA secondary structure is shown in the fourth line. The Base Deep Index (BDI, a number to indicate the relative coverage of each base) of each nucleotide is in the fifth line. The rest lines are the tsRNA reads and alignments. Numbers on the right indicate the number of tsRNA reads. **(B)** RT-PCR amplification of adaptor-ligated *P. sojae* sRNAs. The vertical line indicates the amplified products. Lanes A and B are two technical repeats. **(C)** Clover-leaf view of cloned tsRNAs derived from tRNA-IleAAT. The candidate tsRNA region is highlighted in red. **(D)** Northern analysis of *Phytophthora* tsRNAs. The lower bands (30–38 nt) are the tsRNAs, while the upper bands (larger than 38 nt) are the cognate tRNAs. Ps and Pp are *P. sojae* and *P. parasitica* RNAs pooled from an equal amount of total RNAs of oospores, mycelia, sporangia, cysts and germinated cysts, respectively. **(E)** Processing patterns of tsRNAs. tRNA-SerTGA, tRNA-ArgACG and tRNA-GlnCTG produce 5′, 3′ and both 5′ and 3′ tsRNAs, respectively. The x-axis shows the position of each base in the tRNA.

To identify *Phytophthora sojae* tsRNAs, we surveyed sRNAs from five distinct developmental stages of the *P. sojae* lifecycle, including sexual oospores, asexual mycelia, sporangia, cysts, and germinated cysts. High throughput sequencing led to the identification (see Supplemental Figure S1) of a total of 41 candidate tsRNAs derived from 26 tRNAs (**Figure 1A** and Supplemental Table S1). To confirm the existence of tsRNAs, we performed small-scale manual cloning of tsRNAs in the 15–50 nt RNA fractions (**Figures 1B,C**; Supplemental Table S1). We chose one tsRNA identified by deep-sequencing and one identified by manual cloning to further confirm existence of *P. sojae* tsRNAs. The results showed that both the cloned and sequenced tsRNAs were detected by Northern blots (**Figure 1D**). These three independent assays indicate that the tRNA-derived fragments we detected are real endogenous sRNAs in *P. sojae*. Northern analyses also indicate that the two tsRNAs selected here are conserved in another oomycete pathogen, *P. parastica* (**Figure 1D**). Aligning the tsRNA sequences to their source tRNA sequences indicated that these tsRNAs were derived from the 5′ arm, the 3′ arm or both arms of the tRNA (**Figure 1E**; Supplemental Table S1). We named the tsRNAs by their source tRNAs, for instance, tsRNA-IleAAT is derived from tRNA-IleAAT.

### Stage Specific Accumulation of tsRNAs during *P. sojae* Development

**FIGURE 2:**
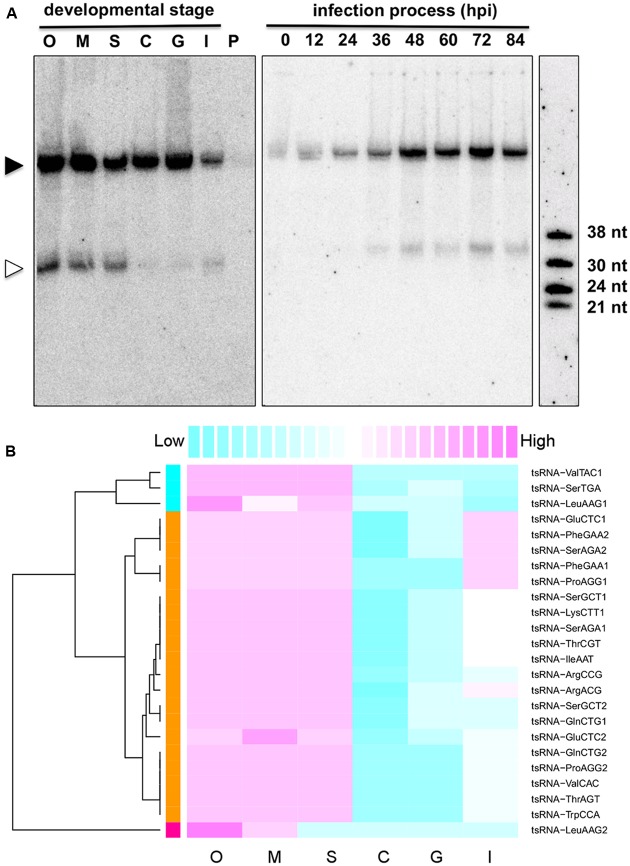
**Stage-specific accumulation of *P. sojae* tsRNAs. (A)** Expression pattern of tsRNA-IleAAT. O, oospores; M, mycelia; S, sporangia; C, cysts; G, germinated cysts; I, infected soybean at 48 hpi; P, uninfected host plant soybean *(G. max)*; hpi, hours post inoculation. Each lane loaded with 20 μg of denatured RNAs. The solid triangle indicates tRNAs, and empty triangle indicates the cognate tsRNAs. **(B)** Conserved expression pattern of *P. sojae* tsRNAs. For each tsRNA, its expression levels among different stages were measured according to the blot intensity. The color-key on the top indicates the relative expression levels of tsRNA, from low (cyan) to high (magenta). Dendrogram on the left illustrates the correlations among the expression patterns of different tsRNAs based on the Pearson distance.

To explore the potential biological roles of tsRNAs, we examined the accumulation of tsRNAs at six different developmental stages of the *P. sojae* lifecycle. The result showed that these tsRNAs had differential levels of accumulation at the different stages (**Figure 2**). For example, tsRNA-IleAAT displayed relatively high levels of accumulation in oospores, mycelia, and sporangia, nearly undetectable levels in cysts and in germinated cysts, low levels upon plant infection (**Figure 2A**, left panel) with gradually increasing levels during plant infection (**Figure 2A**, right panel). Strikingly, such expression patterns were nearly identical for most of *P. sojae* tsRNAs during these development stages (**Figure 2B**, Supplemental Figure S2 and Table S1). The expression levels of tsRNAs relative to cognate tRNAs were highly variable, suggesting that the relative amount of tsRNAs is independent of tRNA abundance (Supplemental Figure S1 and Table S1).

### The Accumulation of tsRNAs and Target Transcripts is Negatively Correlated

**FIGURE 3:**
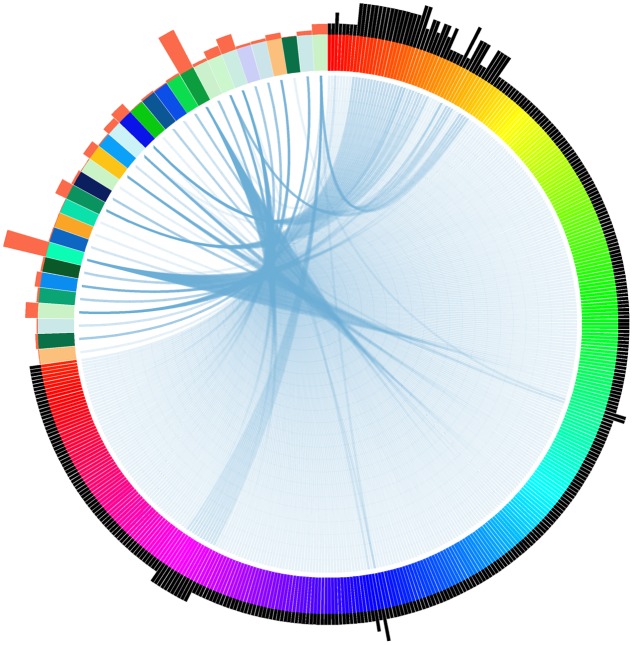
**Relationship between tsRNAs and their putative targets in *P. sojae*.** Each wide color strip on the color circle (the top-left area) represents a single tsRNA, while each narrow color strip on the color circle (the remaining part) represents a target gene. Blue links between wide and narrow color strips represent sequence complementarity between tsRNAs and their predicted targets. The number of tsRNA/target pairs is reflected in the histograms outside the circle: histograms in orange represent the relative number of targets of each corresponding tsRNAs, while histograms in black represent the relative number of tsRNAs that the corresponding targets can bind. The target genes and tsRNAs are arranged clockwise as listed in Supplemental Table S2.

To identify if there are potential homologous target genes of tsRNAs, we searched all available *P. sojae* gene models with their 200 bp flanking sequences for complementary matches to the candidate tsRNAs. A total of 456 complementary sites (Supplemental Table S2), representing 371 putative tsRNA target genes with various functions (Supplemental Table S3), were identified. Notably, we found that one tsRNA could target to multiple genes, and one gene could bind with multiple tsRNAs (**Figure 3**). Among the tsRNA candidates examined in the analysis, 32 had the potential to bind to at least one transcript (**Figure 3**).

**FIGURE 4:**
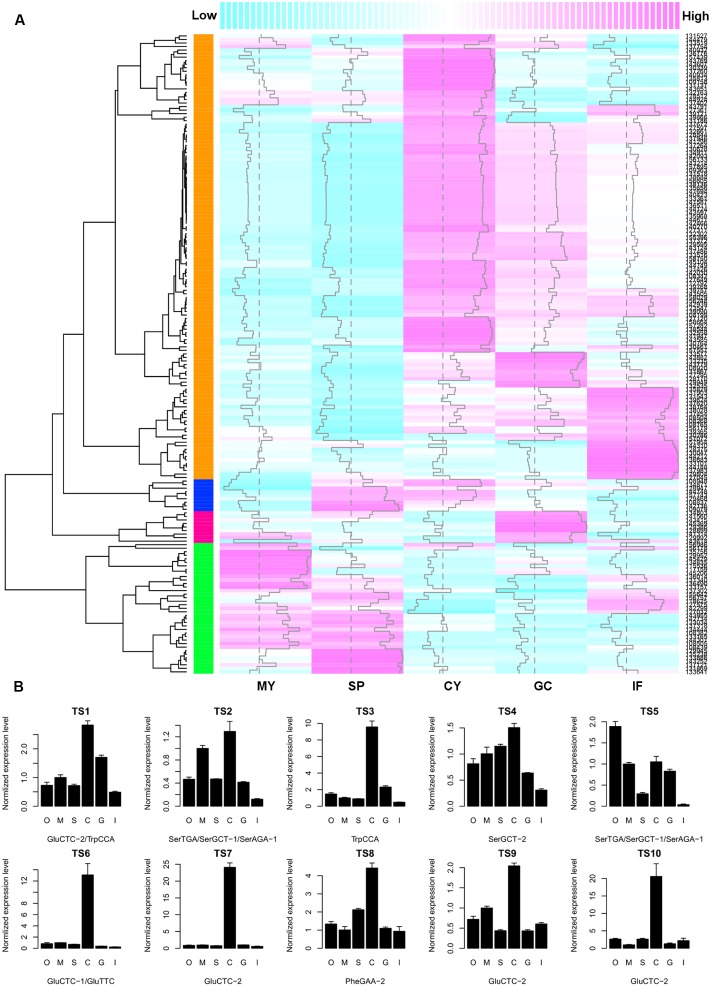
**The negative correlation between tsRNA abundance and target transcript accumulation in *P. sojae*. (A)** Heatmap showing the digital gene expression profile of tsRNA target genes. Column samples MY, SP, CY, GC, and IF, extracted from the *Phytophthora* Transcriptome Database, correspond to the *P. sojae* developmental stages of M, S, C, G, and I as described in this study, respectively. Each row represents one predicted target gene. Dashed vertical line indicates the median of expression levels of all predicted target genes at each of the five developmental stages; vertical trace line inside each column indicates expression level of the corresponding gene. The color-key on the top indicates the relative expression levels of the target genes, from low (cyan) to high (magenta). Dendrogram on the left shows the distance of each two target genes based on their transcript levels. The colored column on the left represent the four clusters of expression patterns of putative tsRNA targets; the cluster in orange color are genes that in general show a negative expression correlation with tsRNA accumulation. **(B)** Quantitative RT-PCR analysis of 10 selected tsRNA putative targets. Error bars indicate the standard deviation of three technical replicates. The bottom text of each barplot indicates the corresponding tsRNA. O, oospores; M, mycelia; S, sporangia; C, cysts; G, germinated cysts; I, soybean infection at 48 hpi. The RNA level in M was set to 1.

To investigate whether there is a negative correlation between tsRNA accumulation and target gene expression, we searched the *Phytophthora* Transcriptome Database (Ye et al., 2011) and obtained digital gene expression profiles of the predicted tsRNA target genes at five developmental stages of *P. sojae*: mycelia, sporangia, cysts, germinated cysts and infection (24-hpi soybean leaves). Since most tsRNAs have a similar and stage-specific developmental expression pattern (**Figure 2B**), a similar but inverse expression pattern of the putative targets was expected. Cluster analysis based on gene expression levels showed that, the biggest cluster of target genes, consisting of 70% (126/181) of the genes analyzed, exhibited abundant transcripts in cysts in which tsRNAs were nearly undetectable, and extremely low transcript levels in mycelia and sporangia where tsRNAs were abundant (**Figure 4A**). Thus, for the majority of the putative target genes, their expression levels correlated negatively with the abundance of tsRNAs. To confirm the profile and negative correlation, we analyzed the expression of 10 putative tsRNA target genes (Supplemental Table S4) using quantitative RT-PCR, and nine of them showed highest expression level in cysts (**Figure 4B**), consistent with the digital expression profile (**Figure 4A**). For example, tsRNA-GluCTC-2 target *P. sojae* gene transcript *TS7*, while tsRNA-GluCTC-2 is absent in cysts, its target *TS7* is highly up-regulated in this stage (Supplemental Figure S3).

### tsRNAs Mediate Degradation of Target Transcripts

**FIGURE 5:**
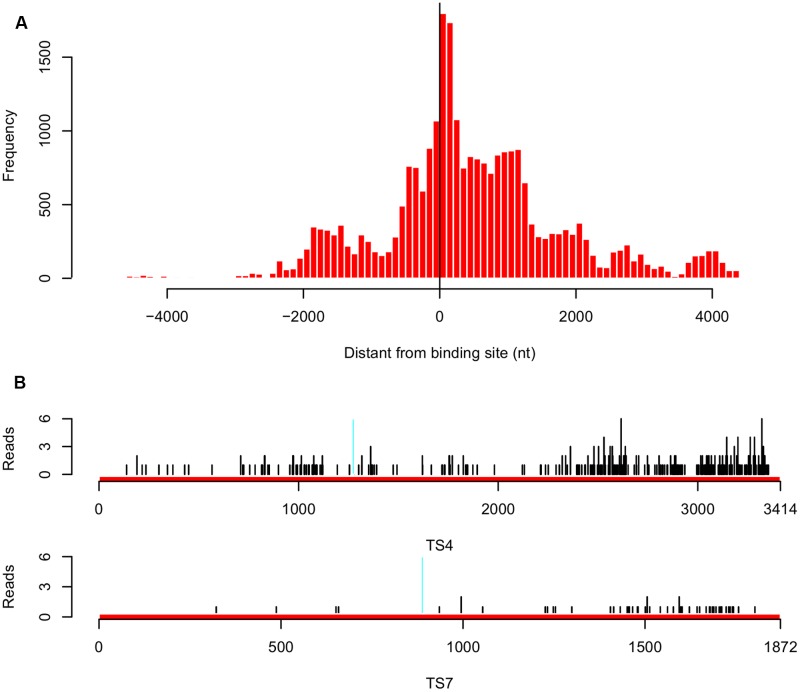
**Analysis of tsRNA-mediated degradation of target transcripts in *P. sojae*. (A)** Distribution of the distance between the degraded reads and target gene binding sites for the 95 target genes that are inversely correlated with accumulation of homologous tsRNAs. “0” in the *x*-axis indicates the degradation event occurred at the center of the binding site, a minus value indicates that in the upstream, while a positive value indicates that in the downstream. **(B)** Distribution of the degradome reads of the tsRNA targets *TS4* and *TS7*. Region in cyan indicates the binding site. Black vertical lines indicate the positions of the degradome reads mapped.

To investigate whether the inverse correlation between tsRNA accumulation and target transcription levels was due to tsRNA mediated mRNA degradation in *P. sojae*, we employed Parallel Analysis of RNA Ends (PARE) technology and sequenced RNA degradome in *P. sojae* mycelia, in which tsRNAs are highly accumulated. We obtained a total of 26,039 reads perfectly matched to 160 predicted tsRNA targets (43% of the total predicted), and 95 of them belong to the biggest cluster (75% of the 126 genes) which were negatively correlated with accumulation of homologous tsRNAs. Most of these 95 genes had fewer matched reads, which were likely a result of tsRNA-mediated degradation. Examining the distances between the tsRNA binding sites and the degradome reads showed that, the degradation events occurred mainly around the binding sites, but extended toward the 3′ direction (**Figure 5A**). Of the 10 genes selected for quantitative RT-PCR confirmation, eight of them had few reads mapped. For example, both *TS4* and *TS7* had a relative many more reads around the binding sites, and many reads were found at the 3′ downstream (**Figure 5B**), indicating that their down-regulation in mycelia is likely due to tsRNA mediated transcript degradation. To verify the degradome sequencing results, we performed 5′ RLM RACE to capture the degraded RNA products in the mycelia of *P. sojae*. Consistent with the degraded reads patterns, we observed that the degradation events occurred around the binding sites with a little bias to the downstream 3′ region (Supplemental Figure S4).

### tsRNA Binding Sites Are Required for Degradation of Target Transcripts in *P. sojae*

**FIGURE 6:**
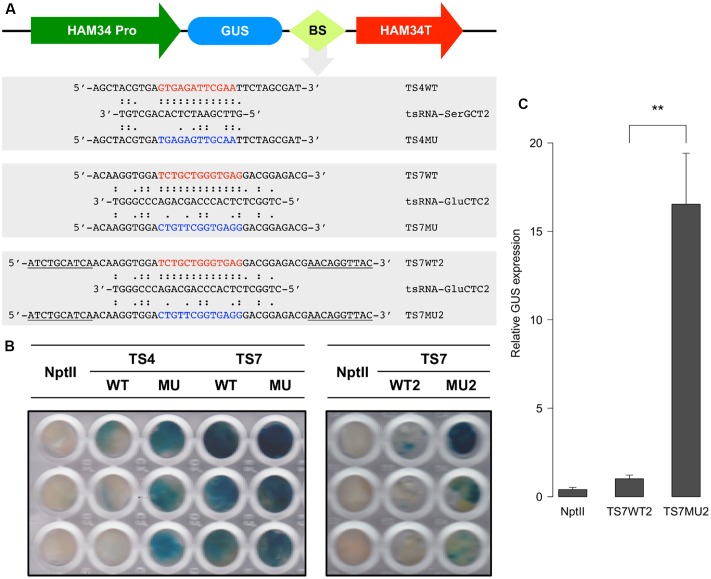
**tsRNA-mediated repression of *GUS* sensor transgene in *P. sojae*. (A)** Structure of the *GUS* sensor constructs used in transient transformation analysis. WT, sensor construct containing wild-type sequence (highlighted in red) of the tsRNA-binding site; MU, sensor construct with a modified sequence (highlighted in blue) of the tsRNA binding sites; BS, tsRNA-binding site. Sequences underline indicate the extended flanking binding sites. **(B)** Histochemical analysis of GUS sensor expression in *P. sojae*. NptII indicate the *P. sojae* cells transformed with NptII plasmid only without GUS sensor constructs, which is used as a negative control here. **(C)** RNA quantitation analysis of *GUS* sensor transcripts in transient *P. sojae* transformants. The *GUS* transcripts detected in TS7WT2 was set to 1, error bars represent the standard deviations from three technique repeats. The significance of difference in *GUS* transcript level between TS7WT2 and TS7MU2 was assessed by *t*-test, “^∗∗^” indicate that the *P*-value is less than 0.01.

The degradome results suggested that tsRNAs mediate RNA degradation in a homology-dependent manner. Since tsRNA is highly expressed in mycelia and is generated from multiple copies of tRNA, it is difficult and unnecessary to knock-down and over-express tsRNA to determine the homology-dependent RNA degradation. To further examine this possibility, we generated sensor constructs containing the β-glucuronidase (*GUS*) reporter gene fused with either the wild-type (WT) or mutant (MU) sequences of the tsRNA-SerGCT2 and tsRNA-GluCTC2 binding sequences of target genes *TS4* and *TS7*, respectively (**Figure 6A**). In the mutant sequences we introduced multiple nucleotide mismatches to disrupt base-pairing with the respective tsRNAs. We transiently transformed *P. sojae* protoplasts with the sensor constructs and determined *GUS* expression using histochemical staining. Almost no blue staining or GUS activity was observed in the independent *P. sojae* cell lines transformed with the TS4WT construct containing the wild-type tsRNA-binding sequence (**Figure 6B**). In contrast, *P. sojae* cell lines transformed with the mutant construct TS4MU showed clear blue staining indicating good levels of GUS expression.

We assayed two sets of GUS sensor constructs for testing tsRNA-GluCTC2-mediated gene silencing, TS7WT, TS7MU and TS7WT2 and TS7MU2. We first prepared and tested the TS7WT and TS7MU constructs containing a short version of the target sequences. The result showed that the WT construct gave very limited decrease in GUS sensor expression in comparison to the mutant construct (**Figure 6B**, left panel). We then considered that sequences flanking tsRNA-binding site could impact the efficiency of tsRNA-mediated gene silencing, and therefore prepared and tested the second set of constructs, TS7WT2 and TS7MU2, containing the tsRNA-binding sites plus 10-nt 5′ and 3′ flanking sequences (**Figure 6A**). This modification dramatically increased the sensitivity of the GUS sensors, and *P. sojae* cell lines transformed with the TS7WT2 construct showed strongly reduced GUS activity in comparison to those transformed with the TS7MU2 construct (**Figure 6B**, right panel). To determine if the repressed GUS activity in the wild-type fusion transgenes is a result of mRNA degradation, we performed quantitative RT-PCR analysis of the transformed *P. sojae* cells. GUS sensor mRNA levels in transformed *P. sojae* cells were much (∼15-fold, *P*-value <0.01) lower with the WT construct than the mutant construct (**Figure 6C**), indicating that GUS sensor repression is associated with RNA degradation. Taken together, results with the GUS sensor constructs indicate that *P. sojae* tsRNAs are capable of triggering RNA degradation-based gene silencing, and suggest that this tsRNA-induced gene silencing requires high-level sequence complementarity between tsRNA and target RNA.

### An Improved High-Quality tsRNA Repository of *P. sojae*

**FIGURE 7:**
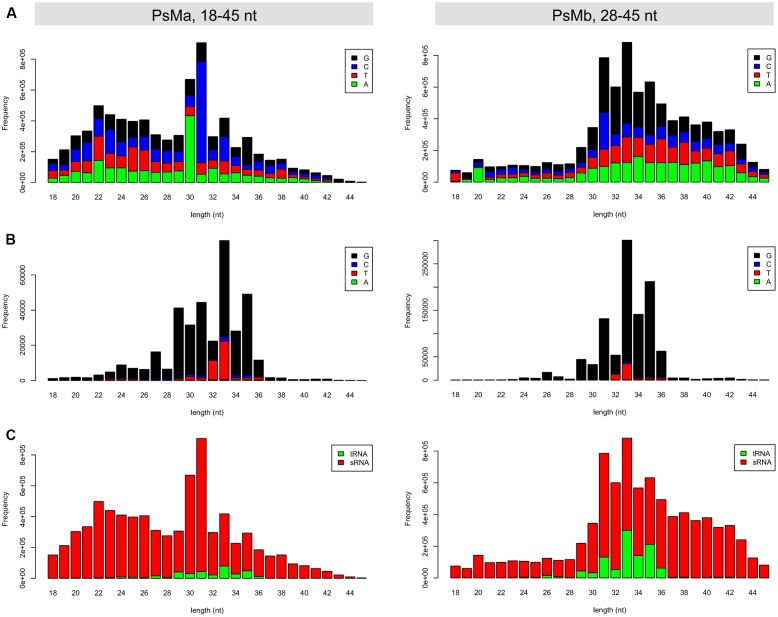
**Length distribution of the re-sequenced mycelial sRNAs in *P. sojae*. (A)** Length distribution and 5′ base preference for the total reads of the 18–45 nt and 28–45 nt sRNAs, respectively. **(B)** Length distribution and 5′ base preference for the tsRNA reads in the 18–45 nt and 28–45 nt sRNAs, respectively. **(C)** Length distribution of the tsRNA and non-tsRNA reads in the 18–45 nt and 28–45 nt sRNAs, respectively. In each of the barplots, *x*-axis represents the size of the sRNA reads, while the *y*-axis represents the frequency of the corresponding sRNA reads.

Owing to the short-range size selection (18–30 nt) in the original sRNA sequencing (**Figure 1A**), the tsRNA dataset used in this study contained mostly the core sequences of tsRNAs but not the full-length size (>30 nt), as expected from the northern blot hybridization results (**Figure 2**; Supplemental Figure S2). To obtain an improved high-quality reference tsRNA sequences, we re-sequenced two sRNA libraries from tsRNA-enriched *P. sojae* mycelia, PsMa and PsMb, with selected sizes ranging from 18–50 nt and 28–50 nt (**Figure 7A**), respectively. In these sRNA datasets, the size of tsRNA reads ranged from 29–36 nt and peaked at 33 nt (**Figure 7B**). Interestingly, the 5′ terminal nucleotides of the tsRNA reads appear to be biased toward G (**Figure 7B**), which is different from miRNA and siRNA which tend to have U at the 5′ end. It is also notable that up to 12% of the total sRNA reads in the range of 28–45 nt were derived from tRNA (**Figure 7C**, right panel).

**FIGURE 8:**
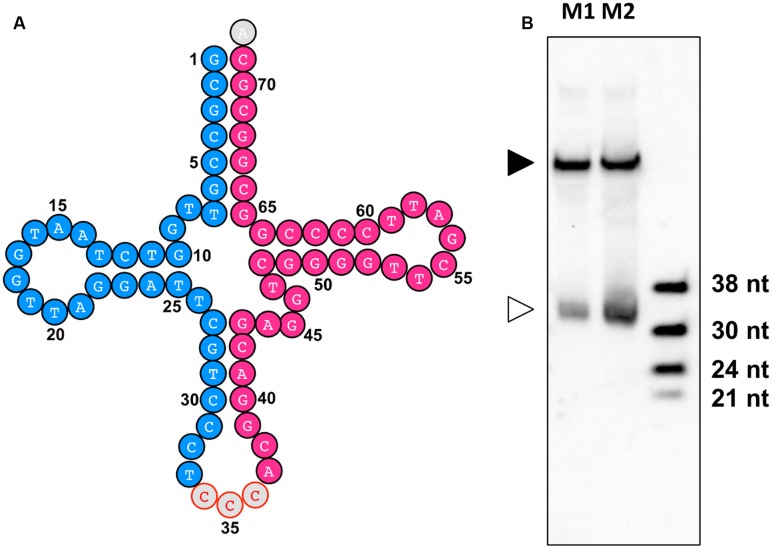
**tsR1 is a 33 nt sRNA derived from tRNA-GlyCCC1. (A)** tRNA-GlyCCC1 generates two tsRNAs. The tsRNA highlighted in blue is tsR1, while the one highlighted in red is tsR18. **(B)** Northern hybridization for tsR1. M1 and M2 are two biological repeats of *P. sojae* mycelial RNAs. The solid triangle indicates tRNAs, and the empty triangle indicates the cognate tsRNAs. Each lane was loaded with 20 μg of denatured RNAs.

By identification of the tsRNAs in these two sRNA libraries, an improved high-quality tsRNA repository was generated (Supplemental Table S5). These tsRNAs are generally highly expressed in the mycelia of *P. sojae*. For example, tsR1 (**Figure 8A**, highlighted in blue), derived from the 5′ arm of tRNA-GlyCCC1, is the most abundant tsRNA in PsMb, accounting for 2.05% of the total reads (Supplemental Table S5). The 3′ arm of tRNA-GlyCCC1, tsR18, was also highly expressed (**Figure 8A**, highlighted in red) in the repository (Supplemental Table S5). To verify if the tsRNAs identified from re-sequencing are full length mature tsRNAs, we performed Northern blot analysis with tsR1. The result is consistent with the size detected by Northern blotting being identical with that detected by re-sequencing (**Figure 8B**).

## Discussion

A variety of sRNAs has recently been identified in eukaryotic pathogens. In fungi, several classes of sRNAs, including miRNA-like RNAs, Dicer-independent siRNAs (Lee et al., 2010), qiRNA, esRNA (Lee H.C. et al., 2009; Nicolas et al., 2010; Nunes et al., 2011b; Chen et al., 2015), and tsRNAs (Jochl et al., 2008; Thompson et al., 2008; Thompson and Parker, 2009; Nunes et al., 2011a) have been identified. However, no canonical miRNAs have been reported (Nunes et al., 2011b). In oomycetes, several miRNAs, a variety of siRNAs, and tsRNAs were discovered recently (Vetukuri et al., 2012; Fahlgren et al., 2013; Qutob et al., 2013; Asman et al., 2014). While miRNAs and siRNAs are known to regulate gene expression, whether and how tsRNAs regulate gene expression remains unclear. In this study, we focused on the recently recognized tsRNA by using soybean pathogen *P. sojae* as a model and identified a large number of tsRNAs in this eukaryote microbe with three independent assays. Our analyses on the targets of these tsRNAs suggest that tsRNAs could induce target transcript degradation in a sequence-specific manner in *P. sojae*.

Previous studies showed that tsRNA have implicated in gene regulation (Haussecker et al., 2010). However, whether sequence homology is required for the tsRNA function remains unclear, as some of the tsRNA regulate the expression of genes that have no homologous target sequence (Ivanov et al., 2011; Sobala and Hutvagner, 2013). In this study, we generated several lines of evidence to suggest that tsRNAs regulate gene expression in a sequence-specific manner. Transcript levels of the putative target genes, predicted based on having 12 to 23 nucleotide perfect complementarity with the tsRNAs, generally showed an inverse correlation with the accumulation of tsRNAs, suggesting that tsRNAs repress the expression of genes due to sequence homology in their RNA transcripts. PARE degradome sequencing and 5′ RLM-RACE experiments showed that RNA degradation sites of target genes were enriched around the tsRNA-binding sites, suggesting that tsRNAs induce target RNA degradation and that the homologous binding sites are important for this tsRNA-mediated RNA degradation. Expression of the GUS sensor constructs containing a WT tsRNA-binding sequence was clearly reduced in comparison to that containing a mutant binding sequence, which again indicates that tsRNAs are capable of inducing target gene repression and that this repression requires sequence complementarity between tsRNA and target RNA. Thus, by using *P. sojae* as a model, we generated compelling evidence indicating a role of tsRNAs in development and supporting an involvement of sequence-specific RNA degradation in the tsRNA function.

Notably, the expression patterns of most *P. sojae* tsRNAs examined were similar, being highly expressed in oospores, mycelia, and sporangia, moderately in germinating cysts and upon plant infection, but nearly undetectable in cysts. This differential accumulation of tsRNAs at different developmental stages and their broadly similar expression patterns suggest the importance of tsRNAs in *Phytophthora* development. In the pathogenic plant fungus *Magnaporthe oryzae*, tsRNAs are more enriched in appressoria than mycelia (Nunes et al., 2011a). In the bacterium *Streptomyces coelicolor*, tsRNA accumulation is temporally correlated with aerial hyphae development (Haiser et al., 2008). These results together suggest that tsRNAs contribute to the development of bacterial, fungal, and oomycete microbes. This is likely a parsimony approach widely employed by microbes for developmental regulation, hitchhiking the genetic selection of their own tRNAs without the need of additional genetic loci.

It is interesting to note that one of the tsRNAs, tsRNA-GluCTC2, targets *TS7* that encodes a translation initiation factor (eIF), making it possible that tsRNA-GluCTC2 plays a role in controlling global protein synthesis. Thus, at the stages where tsRNAs are highly expressed, protein synthesis and metabolism are likely modulated due to three layers of regulation. First, cleavage of tRNAs reduces the efficiency of protein synthesis. Second, the tRNAs cleavage product, tsRNAs, downregulate the abundance of target mRNAs. Third, the reduced amount of proteins, such as TS7, limits the translation initiation process and thus inhibits the global protein synthesis. tsRNAs have been reported to inhibit protein synthesis in other organisms (Zhang et al., 2009; Ivanov et al., 2011; Sobala and Hutvagner, 2013). In *P. sojae*, cyst germination is rapid and important for the early stage of plant infection. The digital expression data show that thousands of genes are up-regulated during cyst germination (Ye et al., 2011). Such a high level of gene up-regulation during cyst germination are also reported for *P. parasitica* (Shan et al., 2004b) and *P. infestans* (Judelson et al., 2008). Thus, pathogen-encoded tsRNAs are likely important for slowing down the turnover of protein synthesis to keep low energy consumption. This inference is supported by the developmental feature of *P. sojae*: in the nutrition lacking stages, namely the oospores, mycelia, and sporangia stages, tsRNAs accumulate at high levels indicating strong cleavage of tRNAs and downregulation of target mRNAs, which can result in low energy consumption; however, at the cyst stage tsRNA accumulation is dramatically reduced, indicating diminished tRNA cleavage and reversal of target gene silencing, which are expected to facilitate successful infection. Once the infection is achieved, the target genes are gradually suppressed, likely to save energy. Consistent with this possibility, tsRNA abundance was shown to increase gradually during the infection process.

In this study, we identified a highly expressed tsRNA, tsR1, which could be used as a model for studying the biogenesis of tsRNA in *P. sojae*. It is interesting that both the 5′ and 3′ tsRNAs of tRNA-GlyCCC1 were highly expressed. According to the positions they located in the cloverleaf secondary structure of tRNA, the biogenesis of tsRNA is DCL-independent in *P. sojae* because tsR1 and tsR18 duplex pair does not form any overhangs. A typical DCL-dependent sRNA always has the 2-nt 3′ overhangs during processing (Elbashir et al., 2001; Lim et al., 2003), and this is also consistent with gene silencing results revealed in *P. infestans* (Asman et al., 2014).

According to our generated results, mechanism underlying tsRNA -mediated target transcript degradation is somehow similar to that of miRNA or siRNA but with distinct features. The most significant difference is in the mode of target gene regulation: miRNA and siRNA usually degraded the target transcripts in the center of the binding sites (Martinez et al., 2002; Yekta et al., 2004), while tsRNAs appear to lead to degradation of the entire transcripts at many sites. Besides, miRNA and siRNA are usually biased to U at the 5′ terminus, while the tsRNAs presented here biased to G at the 5′ ends. Since the 5′ terminus is sensitive to the sorting of AGO proteins (Mi et al., 2008), this result indicates that tsRNAs are likely to use a different AGO protein in their biogenesis or targeting. In a recent study, it was shown that *P. infestans* tsRNAs are AGO1-dependent (Asman et al., 2014). In the present study, we showed that the tsRNAs are differentially accumulated in different tissues, we thus searched the expression pattern of *P. sojae* *AGO1* against *Phytophthora* Transcriptome Database (Ye et al., 2011). Interestingly, we found that the expression pattern of *PsAGO1* (Supplemental Figure S5) is nearly overlapped with that of tsRNA accumulations. Hence, it is quite possible that tsRNAs are associated with AGO1 protein in oomycete pathogens.

In summary, we identified a large number of tsRNAs in the oomycete pathogen *P. sojae*. We found that the tsRNAs accumulated in a similar developmental stage-specific manner, which are negatively correlated with the expression pattern of the predicted target genes. By degradome sequencing and 5′ RLM RACE experiments, we found that tsRNAs could induce the degradation of target transcripts. Finally, by transient expression assays using GUS sensor constructs, we found that the sequence complementarity is critical in tsRNA-mediated RNA degradation in *P. sojae*. Our results show that tsRNAs are a class of functional endogenous sRNAs in oomycetes and they regulate gene expression by inducing sequence-specific degradation of target transcripts.

## Author Contributions

WS, QW, and M-BW conceived the research; QW, TL, JQ, KX, WZ, XW, WJ, MZ, and GF performed the experiment; QW, WS, and M-BW performed data analysis; QW, M-BW, and WS wrote the paper with contributions from all authors. All authors read and approved the final manuscript.

## Conflict of Interest Statement

The authors declare that the research was conducted in the absence of any commercial or financial relationships that could be construed as a potential conflict of interest.

## Footnotes

1 https://github.com/wangqinhu/tsRFinder
