# Supplementary material for: The tRNA-Derived Small RNAs Regulate Gene Expression through Triggering Sequence-Specific Degradation of Target Transcripts in the Oomycete Pathogen Phytophthora sojae
Source: Front Plant Sci. 2016 Dec 22;7:1938. doi: 10.3389/fpls.2016.01938 (PMC5177647; doi:10.3389/fpls.2016.01938)
Supplement: Supplementary file 2 [file Image_1.pdf]

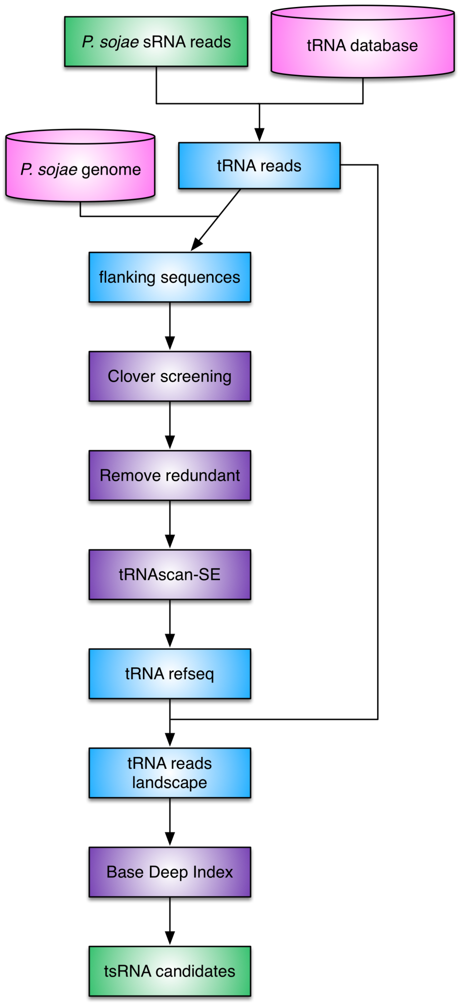

Supplemental Figure S1. Flowchart for tsRNA identification. The tRNA reads were extracted by comparison the *P. sojae* sRNA reads and tRNA sequences. After mapping these reads to the genome, the flanking genomic sequences were extracted for clover-leaf structure screening. After remove the duplicated tRNA sequences, the precise tRNA coding regions were defined by applying tRNAscan-SE. The sRNA reads were mapped back to these reference tRNA sequences and meanwhile, the tRNA reads landscapes were generated. With the aid of Base Deep Index, all tsRNA core sequences identified.

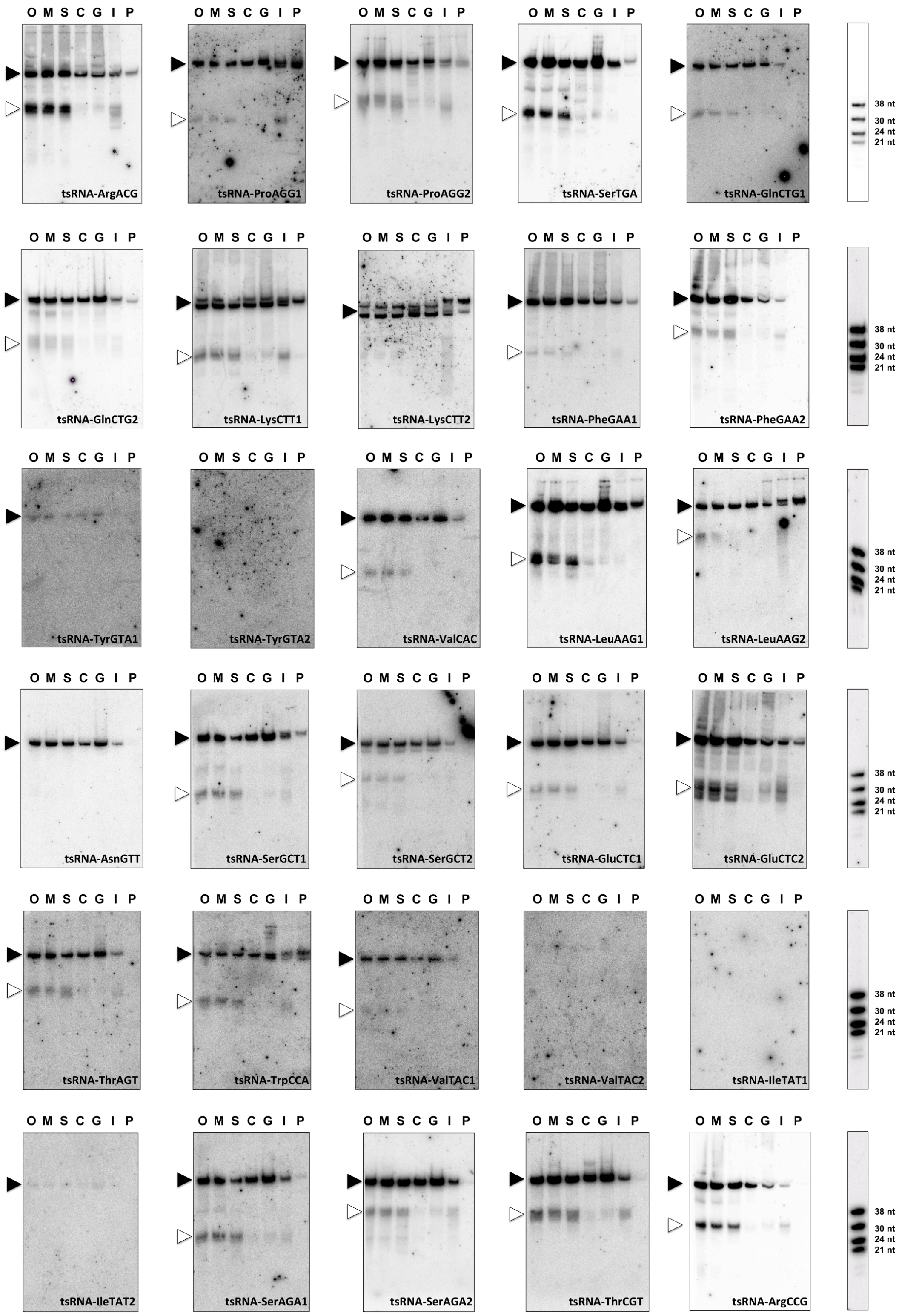

Supplemental Figure S2. Northern blot analysis of tsRNA accumulation at the different stages of *P. sojae* life cycle. O, oospores; M, mycelia; S, sporangia; C, cysts; G, germinated cysts; I, infected soybean at 48 hpi; P, uninfected host plant soybean (*G. max*). Each lane loaded with 20 µg of denatured RNAs. The solid triangles indicate tRNAs, and empty triangles indicate the cognate tsRNAs.

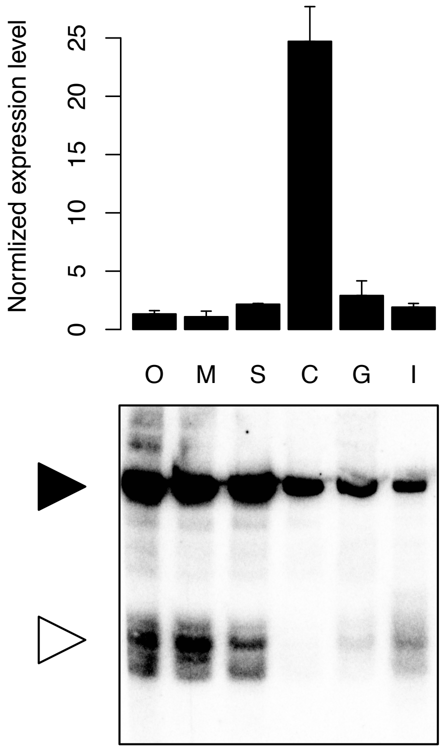

Supplemental Figure S3. The tsRNA-GluCTC-2 accumulation is negative correlated with its target gene *TS7* expression. The expression of *TS7* determined by qPCR (above) and the accumulation of tsRNA-GluCTC-2 determined by Northern (below) are cropped form Figure 4B and Figure S2, respectively. In the qPCR analysis, error bars indicate the standard deviation of three technical replicates. The RNA level in M was set to 1. In the northern analysis, each lane loaded with 20 μg of denatured RNAs. The solid triangles indicate tRNAs, and empty triangles indicate the cognate tsRNAs. O, oospores; M, mycelia; S, sporangia; C, cysts; G, germinated cysts; I, soybean infection at 48 hpi.

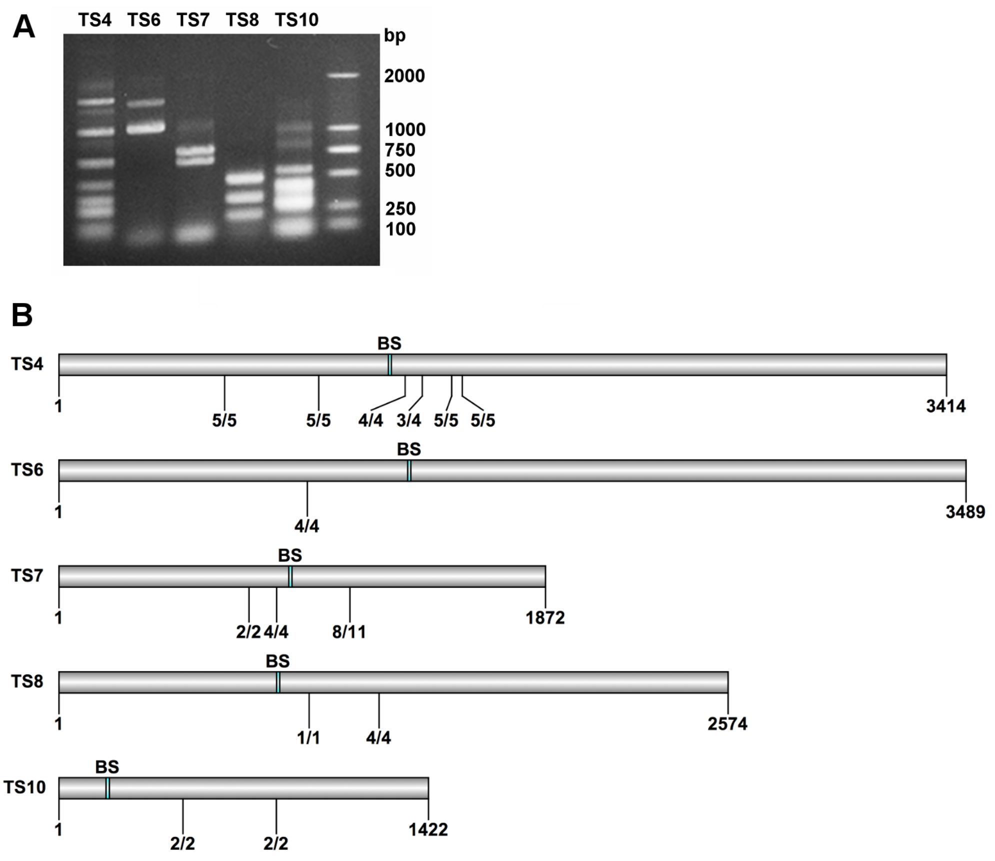

Supplemental Figure S4. 5' RLM RACE analysis on target genes of the tsRNA in the mycelia of *P. sojae*. (**A**) EtBr assay on 5' RLM RACE of mRNA degraded fragments. (**B**) Profile of 5' RLM RACE determined degraded sites. BS stands for the binding site. The numbers of real degraded product clones versus the total clones sequenced were indicated under the genes.

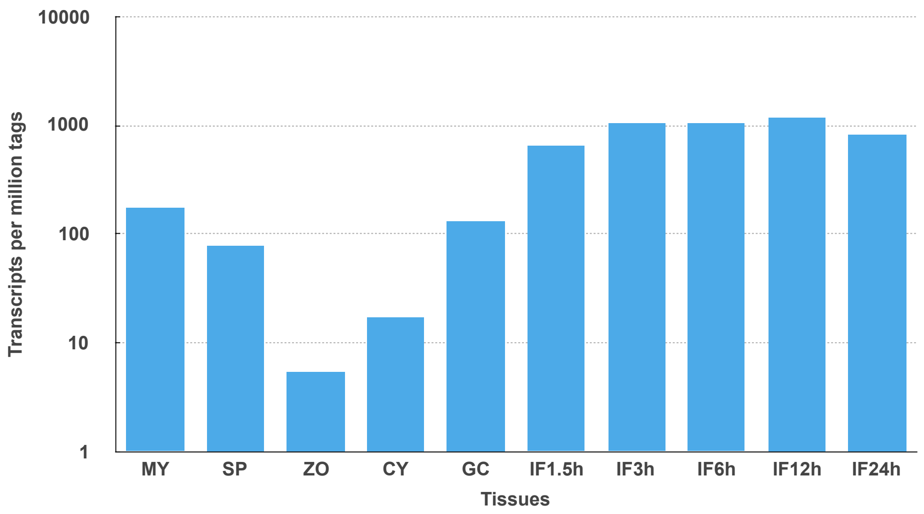

Supplemental Figure S5. The expression pattern of *PsAGO1*. Data are retrieved from the *Phytophthora* Transcriptome Database. MY, mycelia; SP, sporangia; ZO, zoospores; CY, cysts; GC, germinated cysts; IF1.5h, IF3h, IF6h, IF12h, IF24h, soybean infection at 1.5, 3, 6, 12, 24 hours post inoculation on the leaves.
